# Application of Tilapia Skin Acellular Dermal Matrix to Induce Acute Skin Wound Repair in Rats

**DOI:** 10.3389/fbioe.2021.792344

**Published:** 2022-02-14

**Authors:** Kangning Lv, Lei Wang, Xiaoli He, Wenjun Li, Lei Han, Song Qin

**Affiliations:** ^1^ School of Ocean, Yantai University, Yantai, China; ^2^ Yantai Institute of Coastal Zone Research, Chinese Academy of Sciences, Yantai, China; ^3^ School of Life Science, Yantai University, Yantai, China

**Keywords:** tilapia skin, acellular dermal matrix, collagen, wound, repair

## Abstract

Extracellular matrix (ECM) material with good biological activity is essential to simulate cell growth microenvironment, induce cell infiltration and angiogenesis, and promote the repair of large area acute skin wound. In this study, tilapia skin acellular dermal matrix (TADM) was prepared to simulate ECM microenvironment, which can promote substantial area acute wound healing in rats. The main component of TADM is type I collagen, which has good physical and chemical properties, biological activity and cell adhesion. TADM is a form of biomaterial with low immunogenicity, low risk of prion infection and lower economic cost than other related materials such as mammalian collagen biomaterials. Our results show that TADM can guide cell infiltration, angiogenesis, regulate the expression and secretion of inflammatory and skin repair correlated factors to promote tissue healing.

## 1 Introduction

Skin is the largest organ of the human body, and its structure is complex. As the first line of defense of human immunity, skin mainly acts as a barrier to the external environment (Morganti et al., 2019). When the skin is damaged, a large number of pathogens invade, and the wound is easy to be infected, causing the immune response of the body. Therefore, the primary goal of skin wound repair is to restore the structure and function of the wound to the level of normal tissue and accelerate wound healing (Lin et al., 2012). Skin trauma, especially full-thickness skin wound healing, due to the lack of dermis, will lead to difficulty in epithelialization of keratinocytes in the natural epidermis. Therefore, full-thickness skin defects exceeding 4 cm can only be effectively repaired by skin transplantation (Arif et al., 2020). Although autologous skin transplantation has the best repair effect, it brings the risk of infection, secondary injury and pain to the donor site (Goverman et al., 2017). The emergence of skin biomaterials has brought dawn to promote wound healing. Biomedical materials have excellent characteristics such as good biocompatibility and low immunogenicity, which can temporarily replace the existing materials for repairing tissues or organs, fill damaged tissues or organs, and induce cell infiltration, proliferation and function. promote tissue or organ repair and healing (Banerjee et al., 2014).

Collagen is one of the main components of extracellular matrix (ECM). 29 kinds of collagen have been found, among which type I collagen is widely found in skin, tendon, bone and other tissues (Alam and Jeffery, 2019; Lim et al., 2019). It can guide the directional growth of fibroblasts and other cells (Sapru et al., 2021). Because of its excellent biological characteristics such as good biocompatibility, biodegradability and low immunogenicity, it has been selected as one of the important material sources which can be widely used in biomedical tissue engineering. In recent years, biomedical materials prepared from heterologous collagen extracted from animals such as land and sea have been widely used, especially from land mammals such as humans, pigs and cattle. However, due to the risk of zoonotic diseases such as foot-and-mouth disease, mad cow disease and religious constraints such as Islam, as well as high production costs, it is difficult to meet the actual demand. As a result, new sources of marine collagen materials, such as fish, which have a wider range of sources, better safety and lower cost, have become one of the alternatives (Zhang et al., 2016).

Acellular dermal matrix (ADM) is generally based on alkaline solution to extract collagen as the premise of acellular removal of the cellular structure of animal skin tissue, the remaining relatively weak immunogenicity of ECM and dermis collagen fiber reticular cell scaffold structure (Loss et al., 2000; Fitton et al., 2001; Crapo et al., 2011). ECM provides and maintains tissue structure, guides and regulates cell growth and migration, and plays a key role in the specific process of skin repair (Nyström and Bruckner-Tuderman, 2019). A variety of related products have been widely used in biomedical fields such as acute skin trauma such as car accidents or burns, induced bone regeneration, breast repair and dura mater repair, and received good feedback (Fosnot et al., 2011; Magnusson et al., 2017; Wang L.-P. et al., 2021).

Moreover, fish collagen has also been widely studied. Stone et al. compared the effects of acellular fish skin and acellular cowhide on burn repair, it proved that acellular fish skin is better than acellular cowhide (Stone et al., 2021); Chen et al. extracted collagen from tilapia, it can quickly and effectively promote full-thickness wound healing in rats (Chen et al., 2019); Hu et al. extracted marine collagen peptides from tilapia skin can promote wound healing such as deep Ⅱ degree scald (Hu et al., 2017); Zhou et al. used electrospinning technology to prepare tilapia skin collagen nanofibers, which can induce skin wound healing. A variety of studies have shown that collagen-rich fish skin materials have high biodegradability, so we judge that the obtained TADM has a good application prospect in the field of acute wound repair and regenerative medicine.

In this study, tilapia acellular dermal matrix (TADM) was prepared from tilapia skin, and its physical and chemical properties were tested. At the same time, biodegradability and biocompatibility were tested by *in vivo* implantation and cell co-culture, and compared with similar products of fetal bovine skin acellular dermal matrix (FBADM) in the market. In addition, according to its characteristics, acute skin wound repair experiments were carried out in SD rats to verify the biological properties of TADM.

## 2 Materials and Methods

### 2.1 Materials

The tilapia skin was purchased from Hainan Qinfu Foods Co., Ltd (Hainan, China). The fetal bovine skin acellular dermal matrix (FBADM) was purchased from Yantai Zhenghai Biotechnology Co., Ltd (Yantai, China). Female Sprague Dawley (SD) rats (200–220 g) was purchased from Jinan Pengyue Experimental Animal breeding Co., Ltd (Jinan, China). New Zealand White Rabbit (2–2.5 kg) was purchased from Qingdao Kangda Biotechnology Co., Ltd (Qingdao, China). All of the other chemicals were of analytical grade and were used without further purification. All animal producers were conducted in general accordance with the guideline of the Institutional of Animal Care and Use Committee.

### 2.2 Preparation of Tilapia Acellular Dermal Matrix (TADM)

**FIGURE 1:**
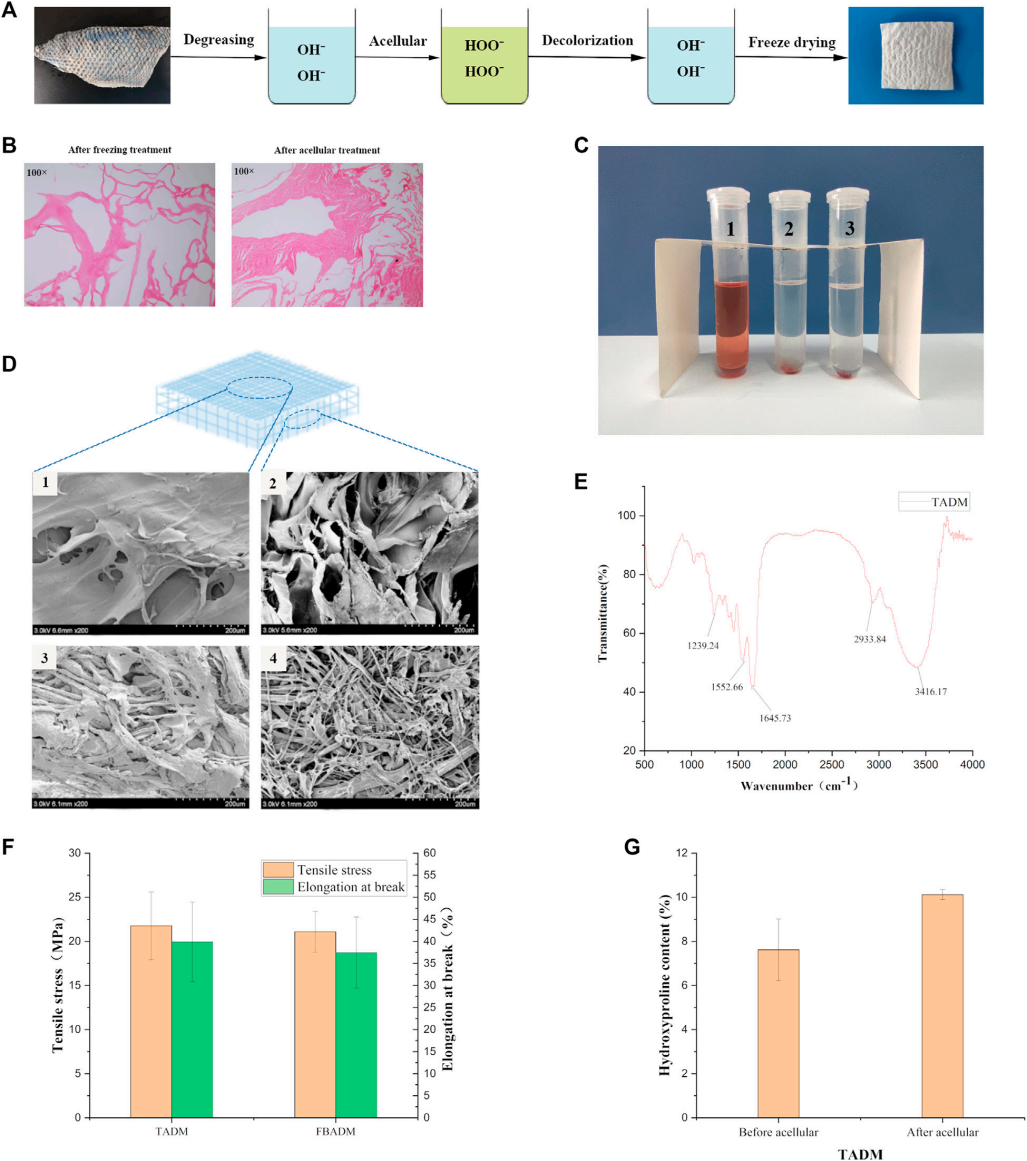
Preparation and characterization of TADM. **(A)** is the flow chart of TADM preparation; **(B)** is the H&E staining map of tilapia skin before and after acellular treatment, the cells in the frozen fish skin have been broken, there are no obvious cellular characteristics, and there is no cell structure at all after further decellularization (Magnification, ×100); **(C)** is the result diagram of TADM hemolysis, C1 is pure water, C2 is normal saline, C3 is TADM extract; **(D)** is TADM and FBADM scanning electron microscope, D1 and D2 are TADM front and side, D3 and D4 are FBADM front and side respectively, TADM and FBDAM front are denser, but FBDAM porosity is higher; **(E)** is TADM’s Fourier transform infrared spectrum (FTIR), amide A/B/Ⅰ/Ⅱ/Ⅲ and other type I collagen characteristic peaks are obvious; **(F)** is the comparison of mechanical properties of TADM and FBADM, and **(G)** is the comparison of hydroxyproline content of tilapia skin before and after acellular. *p < 0.05, **p < 0.01.

Soak the treated tilapia skin in 3% sodium bicarbonate (NaHCO_3_) solution for 12 h with a material-to-liquid ratio of 1:10 (w/v). The fish skin was soaked in 0.1 M sodium hydroxide (NaOH) solution for 8 h and the ratio of material-to-liquid was 1:10 (w/v). Wash with 0.01 M phosphate buffer (PBS) for 3 times to remove fat and other impurities. Then the fish skin was soaked in pure water and 1 m sodium chloride (NaCl) solution for 12 h, and the ratio of material-to-liquid was 1:5 (w/v). The fish skin was soaked in 0.1 M NaOH solution for 6 h, the ratio of material-to-liquid was 1:10 (w/v), and soaked for 6 h. Wash the fish skin with 0.01 M PBS for 3 times and decellularize the fish skin. Then the fish skin was soaked in 3% hydrogen peroxide (H_2_O_2_) solution for 6 h, the ratio of material-to-liquid was 1:5 (w/v), and the fish skin was bleached. Wash with 0.01 M aseptic PBS for 3 times. Finally, clean the TADM (wet) with pure water, cut it to the size of 5 cm × 5 cm, freeze-dry it in a freeze dryer, and sterilize it with ^60^Co (Figure 1A).

### 2.3 Physical and Chemical Properties of TADM

#### 2.3.1 Hematoxylin-Eosin Staining

Tilapia skin and TADM were fixed with 4% (w/v) paraformaldehyde, dehydrated and embedded in paraffin. 5 μm slices were cut, stained with hematoxylin-eosin (H&E) staining solution and observed under ordinary light microscope (DM1000 LED, Leica, Germany).

#### 2.3.2 Hemolysis Test

TADM and aseptic saline (10 mm × 60 mm:1 ml) were extracted at 37°C for 72 h. Blood was collected from the middle auricular artery of New Zealand white rabbits. 2% (w/w) potassium oxalate solution was used as an anticoagulant, and anticoagulant (1 ml rabbit blood: 50 μL 2% potassium oxalate) was mixed with normal saline at the ratio of 4:5. Three tubes of 5 ml TADM extract were prepared as experimental group. At the same time, the same amount of deionized water was used as the positive control group and the same amount of normal saline as the negative control group. The centrifuge tubes of each group were preheated in a water bath at 37°C for 30 min 100 μL diluted anticoagulant rabbit blood was added to each tube, mixed evenly, and 60 min was incubated at 37°C. After incubation, the tubes were centrifuged at 2,100 rpm speed for 5 min in a high-speed centrifuge (Sorvall ST 8R, Thermo, United States). After centrifugation, the hemolysis was observed, and the absorbance of the supernatant of each group was observed under visible spectrophotometer (UV2450, Shimazu, Japan) 545 nm, which was adjusted to zero with normal saline. The hemolysis rate is calculated according to the following formula.

$$Hemolysis\;rate\;\left(\text{\%}\right)=\frac{\text{A}1-\text{A}2}{\text{A}3-\text{A}2}\times 100\text{\% }$$ (1)

In the formula, A_1_ is the average absorbance of TADM group, A_2_ is the average absorbance of normal saline group, and A_3_ is the average absorbance of deionized water group.

#### 2.3.3 Scanning Electron Microscope

The structures of TADM and FBADM were observed by scanning electron microscope (SEM). The TADM was cut into small pieces of 1 mm × 1 mm and fixed on the sample table by conductive adhesive. After the surface was sprayed with gold, the microstructure of the TADM or FBADM was observed by Smur4800 cold field emission SEM (S-4800, HITACHI, Japan).

#### 2.3.4 Determination of Porosity

TADM was cut into 10 mm^2^ sized tissue and put it in a 10 ml pycnometer filled with anhydrous ethanol, and weigh the weight of the pycnometer before and after putting into the acellular tissue. Measure the total porosity (P) of TADM according to the following formula:

$$Porosity\left(\%\right)=\frac{\text{W}2-\text{W}1}{\text{W}1}\times 100\text{\%}$$ (2)

In the formula W_1_ refers to the weight of the pycnometer before it is put into the tissue, and W_2_ is the weight of the pycnometer after it is put into the tissue.

#### 2.3.5 Determination of Swelling Ratio

Swelling ratio is an important index to evaluate the hydrophilicity of wound dressing agents, because higher swelling rate is beneficial to cell adhesion and osmotic growth (Amirian et al., 2021; Tan et al., 2021; Wang S. et al., 2021). First, weigh the dry weight (D) of three samples (100 mg) TADM and FBADM scaffold, soak them with pure water, remove the surface water of the scaffold with filter paper, weigh the scaffold wet weight (W), and calculate the swelling rate of the acellular scaffold with the following formula:

$$Swelling\;rate\;\left(\%\right)=\frac{\text{W}-\text{D}}{\text{D}}\times 100\text{\%}$$ (3)

In the formula, W is the wet weight and D is the dry weight.

#### 2.3.6 Determination of Fourier Transform Infrared Spectroscopy

TADM and FBADM were cut to the size of 1 mm × 1 mm, crushed and used for infrared spectroscopy. The infrared spectrum of collagen in the scaffold was recorded by Fourier transform infrared spectroscopy (Nicolet 6700, Thermo Fisher, United States). The scanning range was 400–4000cm^−1^. The data was analyzed by Origin 2021 (MicroCal, United States) software. The absorption peaks of collagen are amide A, amide B, amide I, amide II and amide III.

#### 2.3.7 Determination of Mechanical Strength

In order to evaluate the mechanical strength of the scaffold, TADM and FBADM with 0.6 mm thickness (measured by spiral micrometer) were cut into long strips of 3.5 cm × 0.2 cm at room temperature, and the tensile strength was tested by universal material testing machine (CMT8502, MTS systems, China). Put the sample in the fixture, the maximum load is 500 cN, the sensitivity 0.01 cN, the test speed 5 mm/min. The number of repetitions of all tests was 5, and the average value was taken.

#### 2.3.8 Determination of Hydroxyproline Content

Hydroxyproline (Hyp) is the main component of collagen, and it’s content can represent the content of collagen. Three samples of 0.2 g freeze-dried tilapia skin and freeze-dried acellular tilapia skin were extracted with 6 M HCl at 110°C. The Hyp content of tilapia skin before and after decellularization was determined by hydroxyproline determination kit (Solarbio, China).

### 2.4 In vivo Degradation

#### 2.4.1 Implantation in Rats

Fifteen SPF-grade 8-weeks female SD rats were selected. After anesthesia, the full-thickness skin openings of 0.5 cm were opened at 1.5 cm on both sides of the dorsal spine, and 0.5 cm × 0.5 cm-sized FBADM and TADM were placed on the left and right sides, respectively, and sutured.

#### 2.4.2 Hematoxylin-Eosin Staining

After the establishment of the model, three rats were taken from each of day 3, 7, 14, 21, and 28 days after the establishment of the model, and the subcutaneously implanted scaffold tissue was removed and fixed in 4% paraformaldehyde solution. After 24 h of fixation, the tissues were embedded in paraffin and sectioned in routine paraffin for HE staining.

After hematoxylin and eosin staining, the morphology and cell infiltration of TADM and FBADM were observed under light microscope. H&E staining can identify the tissue cell structure and its internal DNA and protein location. DNA is dyed blue or purple and protein is dyed red or pink.

### 2.5 Cell Culture

In order to evaluate the cytotoxicity, proliferation rate and adhesion of TADM, we chose mouse fibroblasts (L929) as co-cultured cells with TADM and FBADM.

#### 2.5.1 Cytotoxicity and Proliferation Rate

Under aseptic condition, TADM and FBADM were cut into 30 mm × 90 mm size and placed in 9 ml Dulbecco modified Eagle high glucose (DMEM-H) complete medium and extracted at 37°C for 24 h to obtain cell culture medium (Ma et al., 2020).

The cytotoxicity was detected by Calcein-AM/PI double staining and CCK-8 method. When the cells grew to the logarithmic phase, the cells were counted by cell counter (TC20, BioRad, United States). According to the standard of 1×10^5^ cells per well, 500 μL cell suspension was put into 48-well plate. There were three parallel groups in the control group and three parallel groups in the experimental group. After the cells adhered to the wall, the original cell culture medium was replaced with the extract. After incubating 15 min at 37°C, the cells were stained with Calcein-AM/PI double staining according to the instructions of the kit (Solarbio, China), and the cells were incubated at 37°C. Fluorescence was excited by 490 nm wavelength under inverted fluorescence microscope (ECHO RVL-100-G, United States), and the distribution of living dead cells was observed. According to the standard of 5×10^3^/well, the mixture of 200 μL cell suspension and extract was sequentially added to the 96-well plate according to the proportion of 20, 40, 60, 80 and 100%. After co-culture with cells for 24 h, each well was equipped with 100 μL CCK-8 solution (10 μL CCK-8 in 90 μL medium) according to the instructions of CCK-8 kit. Then the culture plate was incubated at 37°C and 5% CO_2_ for 2 h, and the opticaldensity(OD) value was detected at 470 nm by microplate reader (Epoch, BioTek, United States).

#### 2.5.2 Observation of Cell Adhesion

L929 cells were cultured in DMEM high glucose complete medium containing 10% fetal bovine serum (Gibco, United States) and 1% penicillin and streptomycin (Solarbio, China). L929 cells were cultured in a cell incubator at 37°C and 5% CO_2_. The cells were digested and counted when the cells grew to the logarithmic phase. After 200 μL cell suspension was inserted into a 96-well plate (2 mm × 2 mm size). TADM and FBADM were put into the cell suspension, co-cultured with the cells for 24 h, fixed with 2.5% glutaraldehyde solution for 24 h, and lyophilized. The infiltration and growth of cells were observed by scanning electron microscope.

### 2.6 Rat Model of Skin Trauma

#### 2.6.1 Establishment of Skin Trauma Model

Eighteen SPF-grade 8-week-old female rats (200–220 g). Aseptic operation was maintained during the operation. 10% chloral hydrate was injected intraperitoneally according to 0.4 ml/kg body weight ratio. Two full-thickness wounds with 1 cm diameter were made on both sides of the dorsal spine of SD rats. One side of the wound was affixed to the wound with TADM or FBADM to avoid infection. The other wound was used as the control group, and the control group did not do any treatment (only medical gauze). Normal feeding after operation. After examining the shape of the wound on the third, 7th and 14th day after operation, three rats in each group were euthanized, and the wound tissue was removed, fixed in 4% paraformaldehyde for 24 h, embedded in paraffin, and randomly cut into 5 μm thick sections.

#### 2.6.2 Histopathological Examination

The tissue sections of the skin taken on the third, 7th and 14th day were stained with hematoxylin-eosin (H&E) and Masson. The histopathology was examined under light microscope. The changes of wound re-epithelialization, fibroblast content, revascularization, inflammatory cells and collagen deposition were observed. The collagen volume fraction was analyzed by ImageJ (National Institutes of Health, United States) software and Origin 2021.

#### 2.6.3 Immunohistochemistry

After the skin tissue samples taken on the 14th day were dewaxed and rehydrated, 30 min was incubated with 3% H_2_O_2_ at 37°C. The slices were washed with PBS for 3 times and 5 min each time. These slices were put into 10 mM citric acid buffer (pH = 6.0), boiled and heated for 5 min, then cooled at room temperature to repair the antigen. It was washed 5 min with PBS for 3 times. Then 5% goat serum was used to seal the non-specific binding site at 37°C for 10 min. After removing the excess liquid, the non-specific binding site was incubated with anti-epidermal growth factor (EGF) antibody, anti-fibroblast growth factor (FGF1) antibody, anti-nerve growth factor (NGF) antibody and anti-Platelet endothelial cell adhesion molecule-1(CD31) antibody at 37°C for 2 h. It was washed 5 min with PBS for 3 times. Then 30 min was incubated with biotinylated HRP horseradish goat anti-rabbit secondary antibody at 37°C. It was washed 5 min with PBS for 3 times. The slices were stained with DAB solution, rinsed with tap water and re-stained with hematoxylin, then the slices were gradient dehydrated and sealed, and the staining was observed under ordinary light microscope.

### 2.7 Statistical Analysis

SPSS25.0 software was used for statistical analysis. The data of all results are expressed as mean ± standard deviation (mean ± SD). Double-tailed *t*-test or one-way analysis of variance (ANOVA) was used for statistical significance. The difference was significant (*p* < 0.05), and the difference was extremely significant (*p* < 0.01).

## 3.Result and Discussion

### 3.1 Physicochemical Properties of TADM

Skin wound repair is a close dynamic process, including inflammation, proliferation, vascularization and regeneration. The repair process not only requires a large number of fibroblasts to proliferate, but also the formation of blood vessels and nerves is equally important. Large area full-thickness skin repair, due to the lack of vascularization of new skin, affecting the effect of repair, resulting in slow wound healing, obvious wound contraction and the formation of obvious scar tissue (Lin et al., 2021). Appropriate ECM structure can provide structural support for the growth and migration of fibroblasts, and combine with it to promote the expression of related factors and promote wound healing (Xue et al., 2021). As a tissue engineering scaffold for wound dressing regenerative medicine, it must have the characteristics of good porosity, mechanical strength, biocompatibility, biodegradability and low immunogenicity. Freeze-drying technology is an important technology for the manufacture of controllable porous structural materials in the application of regenerative medicine. Porous spatial structure and tissue cell microenvironment compatibility are very important for wound repair materials, because they are conducive to cell infiltration, growth and proliferation, and free transport of other nutrients. The swelling ability of biomaterials plays an important role in the cascade of hemostasis and wound healing by controlling the ability of drug release, degradation, blood immersion and water retention at the wound site. Freeze-dried tilapia acellular dermal matrix (TADM) is a natural ECM structure that can soak and absorb large amounts of water or biological liquids. After calculation, the porosity of the TADM support can reach 82.51%, the swelling rate is as high as 1,549%, and the hydrophilicity and water retention are very good. It is beneficial to cell infiltration and growth. Mechanical strength is another important index to evaluate the mechanical properties of scaffolds, because the higher mechanical strength is conducive to maintaining the integrity of scaffolds structure.

Figure 1A shows that the freeze-dried TADM is porous and spongy. The cell structure of frozen tilapia skin could not be seen after HE staining (Figure 1B, left). By acellular treatment, the cells in TADM were completely removed and the structural integrity of collagen fibers was not damaged (Figure 1B, right). The results of hemolysis test of New Zealand white rabbits showed that TADM material did not have hemolytic properties (Figure 1C). As shown in Figure 1D, the results of scanning electron microscope show that both TADM and FBADM have high porosity and their structures are different. TADM can be divided into dense layer (outer surface) and loose layer (inner layer), and the pore structure is mainly thin film lamellar structure. On the other hand, both the front and side of FBADM show pore structure. The surface dense structure is more beneficial to block the contact between the wound and the external environment.

Figure 1E shows the infrared spectrum of acellular dermal matrix of tilapia skin, and the typical peaks of type I collagen: amide A, amide B, amide Ⅰ, amide Ⅱ and amide Ⅲ can be seen. In the infrared band of type Ⅰ collagen, the amide A absorption peak appears in the range of 3,400–3,440 cm-1, and the peak shifts to a lower frequency when the -N H group of collagen peptide participates in the formation of hydrogen bond; the characteristic absorption frequency of amide Ⅰ band is between 1,600 and 1700 cm-1; the normal absorption range of amide Ⅱ band is between 1,500 and 1,600 cm-1; the absorption range of amide Ⅲ peak is between 1,200 and 1,360 cm-1 (Li et al., 2013). Therefore, the infrared spectrum results show that the main component of TADM is type I collagen, and the collagen fiber triple helix structure is complete, which is very suitable for use as regenerative medicine materials. Figure 1F shows that the average maximum tensile forces of TADM and FBADM are 21.76 and 21.09 MPa, respectively, and the average elongation at break is 39.89 and 37.47%, respectively, indicating that TADM has excellent mechanical strength and is very suitable for biomedical materials. Figure 1G shows that the average hydroxyproline content of tilapia skin before and after acellular treatment is 7.62 and 10.12% respectively, and there is little difference between them, indicating that acellular treatment has little effect on TADM collagen content.

### 3.2 Biocompatibility Evaluation of TADM

**FIGURE 2:**
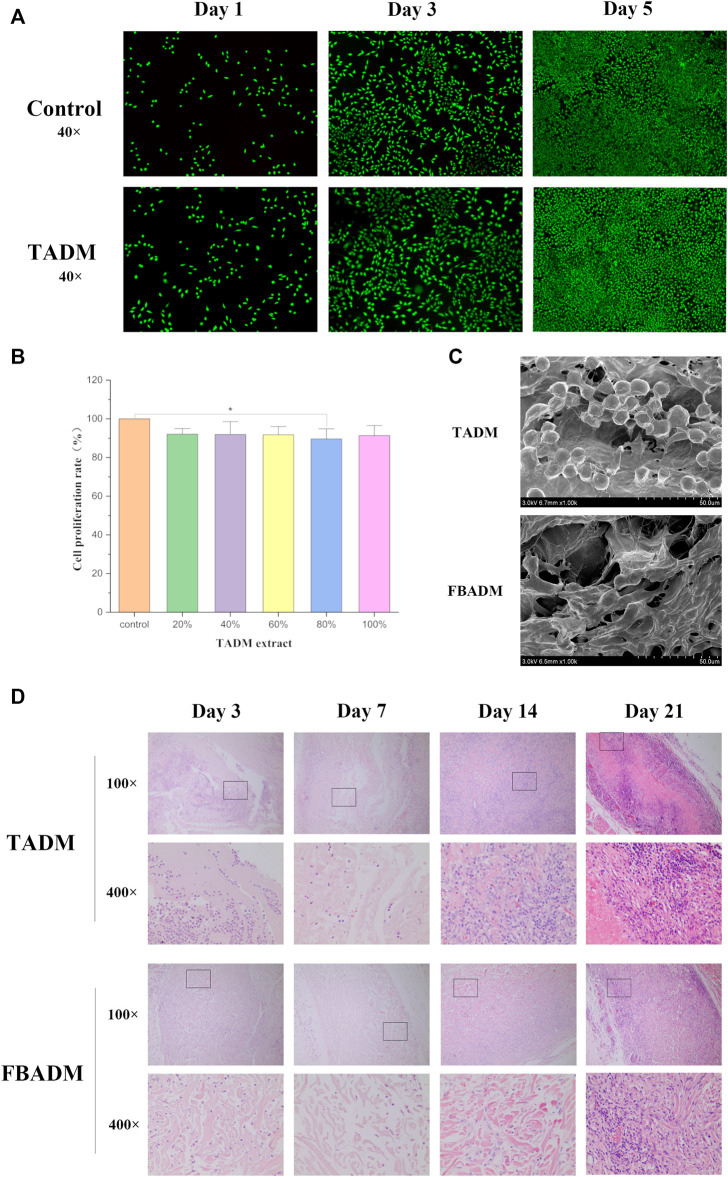
Biocompatibility evaluation of TADM. **(A)** is the TADM extract to culture L929 cells on the first, third and fifth day of AM/PI staining (green for living cells and red for dead cells; magnification, ×40); **(B)** is the picture of CCK-8 to detect the effect of different concentrations of TADM extract on the proliferation rate of L929 cells; **(C)** is the electron microscope picture of the adhesion and growth of L929 on TADM and FBADM; and **(D)** is the H&E staining map of degradation of TADM and FBADM *in vivo*. The tissues were taken for H&E staining on the third, seventh, 14th and 21st day after implantation (magnification, ×100, 400×). *p < 0.05, **p < 0.01.

In order to confirm the biocompatibility of tilapia acellular dermal matrix (TADM) materials, we verified them *in vitro* and *in vivo*. Cytotoxic materials can affect cell metabolism. After the Calcein AM-PI staining, the living cells were green and the dead cells were red (Zhang et al., 2021). Figure 2A shows that both the control group and the TADM group have obvious proliferation trend within three and 5 days. The experimental results show that the cells in the TADM group grow well compared with the control group, and there is no significant difference in the proportion of dead cells, indicating that the TADM scaffold has no cytotoxicity. Similarly, the results of CCK-8 test (Figure 2B) also proved that there was no difference in cell proliferation rate and cytotoxicity between the TADM group and the control group, and the biocompatibility of TADM was satisfactory.

Figure 2C shows that L929 cells can normally infiltrate TADM and adhere to it. Like FBADM, TADM has good fibroblast adhesion and potential to induce fibroblast migration and growth. The results of cytological cell adhesion also show that TADM not only has high porosity, but also facilitates cell adhesion and is friendly to cell growth.

Through subcutaneous implantation in rats, it was found that TADM had tissue fluid infiltration and adhesion to the surrounding tissue on the third day, and gradually degraded to form a tissue mass on the seventh/14th day. On the 21st day, part of the matrix was basically degraded, and on the 28th day, the TADM was completely degraded and the samples could not be obtained. This is basically the same as the degradation rate of FBADM samples *in vivo*. The results of *in vivo* degradation experiment in rats showed that there were no adverse reactions such as allergy in all rats during implantation.

The histological staining of subcutaneously implanted fish skin acellular dermal matrix is shown in Figure 2D. The results show that TADM and FBADM have a large amount of tissue fluid infiltration on the third day, some inflammatory cells enter the scaffold along the larger pores, the structure of the scaffold is intact, and the fiber coating begins to form. On the seventh day, a small number of inflammatory cells in the scaffold infiltrated into the smaller pores of the scaffold, and the collagen fibers in the scaffold began to degrade, which means that the degradation has begun, and the scaffold has adhered to the skin connective tissue. On the 14th day, the scaffold has been completely infiltrated by macrophages and other inflammatory cells, forming a large number of multinucleated macrophage phagocytic scaffolds, and the collagen fiber structure is not intact, which is basically integrated with the skin and cells like normal soft tissue. On the 21st day, the structure of the scaffold could not be distinguished, and most of the inflammatory cells were plasma cells, which formed chronic inflammation and were adapted by the body. With the extension of implantation time, more and more inflammatory cells were immersed into the scaffold, phagocytosis and degradation of the scaffold.

### 3.3 Skin Wound Repair in Rats

The safety and efficacy of the obtained tilapia acellular dermal matrix (TADM) were evaluated by full-thickness skin wound healing in rats for 2 weeks, and compared with negative control and positive control commercial scaffold (FBADM).

**FIGURE 3:**
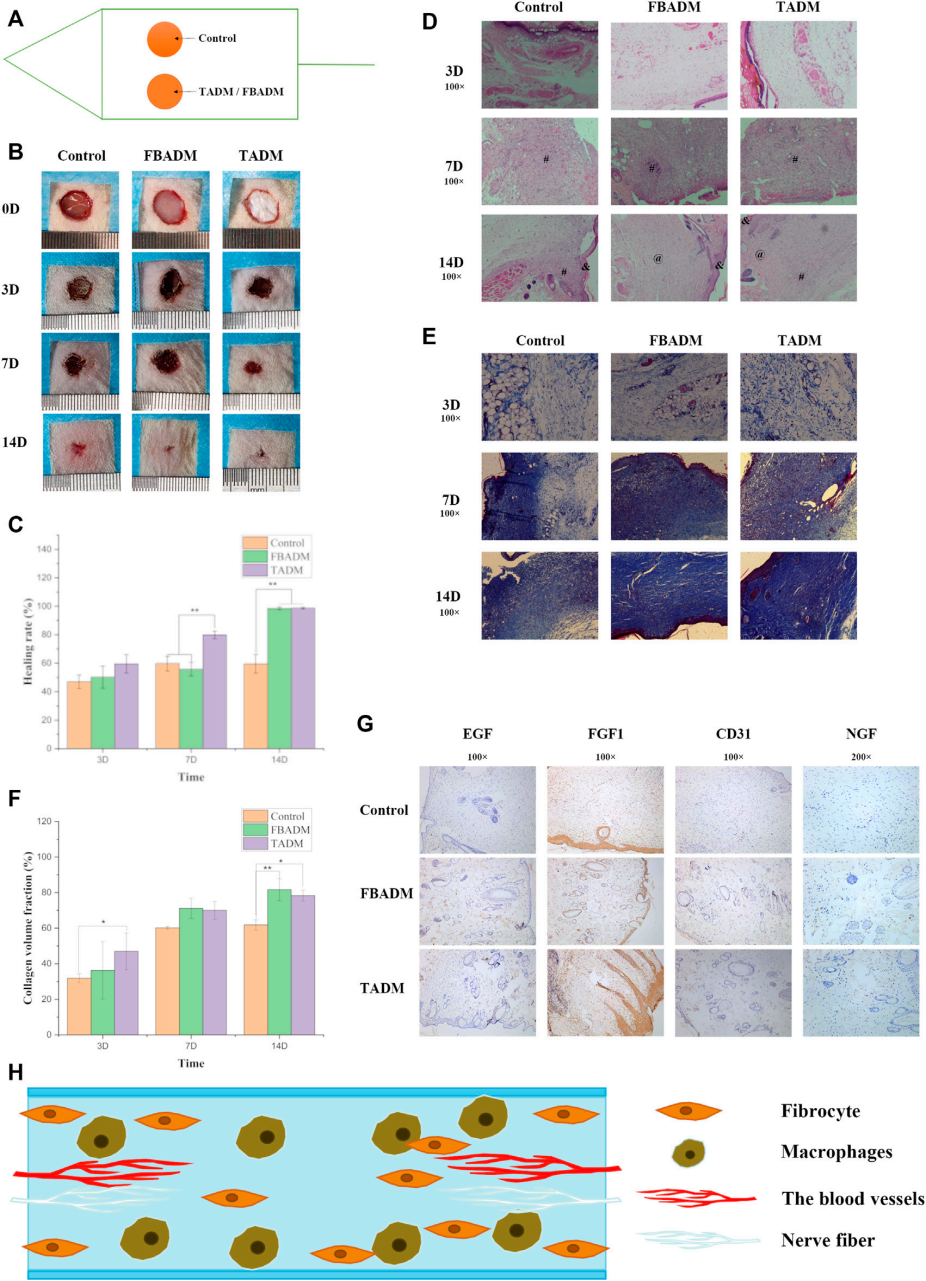
TADM on acute skin wound repair in rats. **(A)** is the schematic diagram of rat wound repair model; **(B)** is the control map of wound healing of TADM, FBADM and control group; **(C)** is the comparison of wound healing rate among groups; **(D)** is the H&E staining map of rat skin section skin trauma, & represents the epidermis,at represents the dermis and # represents the granulation tissue (Magnification, ×100); **(E)** is the Masson staining map of rat skin section skin trauma (Magnification, ×100); **(F)** is the comparison of collagen volume fraction among groups; **(G)** is the immunohistochemical picture of rat skin section on the 14th day of skin trauma (The expression and secretion of EGF/FGF1/NGF/CD31 factor significantly increased, Magnification, ×100; 100×; 100×; 200×); and **(H)** is the model map of skin repair induced by TADM. *p < 0.05, **p < 0.01.

Figures 3B,C shows that the wound in each group shrank significantly in the first 3 days, and the extent of reduction was similar, but the control group had obvious sunken scab, while the cowhide texture was hard, which made the blood scab form a large fold, which was not beautiful and easy to rupture, resulting in secondary injury. On the seventh day, the wound in the TADM group was significantly smaller than that in the FBADM group and the control group, and there was no significant difference in healing area between the FBADM group and the control group. On the 14th day, the wounds in the TADM group and the FBADM group basically healed, while the control group still did not completely heal and formed a large scar. The degree of redness and swelling in the TADM group and FBADM group was significantly slighter than that in the control group, and there was no visible infection or immune rejection in the whole repair process, which indicated that TADM had good isolation ability and anti-bacterial infection ability to the wound and external environment, and good biocompatibility.

Histomorphological analysis of regenerated skin tissue in the healing stage was used to evaluate the therapeutic effect of TADM on wound healing. Epithelialization is a decisive parameter for successful wound closure. In the absence of epithelialization, the wound cannot be considered to heal. H&E staining (Figure 3D) showed that at the initial stage of repair, inflammation occurred in the control group, TADM and FBADM groups, which resisted the invasion of external pathogenic microorganisms and allograft rejection, which belonged to the normal body immune phenomenon. After 7 days, a large number of newly formed dermis and granulation tissues were formed near the wound in FBADM and TADM groups, which proved that the repair speed was accelerated to the 14th day. The wounds in the FBADM and TADM groups were basically repaired by newly formed dermis, and only a small amount of granulation tissue existed, while there was still more granulation tissue in the control group. The results showed that mature blood cells, hair follicles, epidermis thickening and fibroblasts could be seen in TADM group at 14th days. TADM had good biological activity. HE staining showed a large number of scar tissue in the wound repair site of control group, while FBADM group and TADM group began to regenerate hair follicles and began to form normal dermal connective tissue. Collagen deposition can be used to evaluate the effect of wound healing. Figures 3E,F showed that on the third, 7th and 14th day of wound repair, the collagen volume fraction in the TADM group was significantly higher than that in the control group.

The immunohistochemical results were shown in Figure 3G, which showed that TADM group and FBADM group significantly promoted the expression of skin-related healing growth factors of EGF, FGF, NGF and CD31. EGF can promote the proliferation and migration of keratinocytes and accelerate epithelialization (Shao et al., 2021). Type I procollagen gene can limit the collagen deposition of scar fibroblasts. FGF can promote the migration, proliferation and vascularization of fibroblasts, down-regulate the expression of type I procollagen gene and prevent scar formation (Xie et al., 2008; Shi et al., 2013). NGF can promote the growth and differentiation of neurons and promote nerve repair. EGF and FGF play an important role in wound healing (Chen et al., 2019). CD31 is a platelet endothelial cell adhesion molecule-1, which represents the proliferation of blood vessels. The relative number and distribution of platelet endothelial cell adhesion molecule-1 in the process of wound healing are of great significance to wound healing. On the 14th day, the expression of EGF, FGF and NGF in TADM group and FBADM group was higher than that in control group. The results showed that TADM has softer texture than similar FBADM sold in the market, TADM promoted the expression of skin, vascular and nerve repair factors such as EGF, FGF, NGF and CD31. TADM can induce and promote the invasion, growth and proliferation of skin fibroblasts, significantly promote wound contraction and healing, promote skin structure reconstruction, prevent wound infection, promote angiogenesis and collagen deposition, and has a better therapeutic effect on full-thickness skin wounds. Because TADM has good tissue space structure, biological activity and mechanical properties, it is very suitable for skin and other soft tissue wounds. These results show that TADM is a promising full-layer skin repair dressing.

## 4 Conclusion

In this study, we prepared an acellular dermal matrix scaffold derived from tilapia skin and characterized its properties. The results show that our TADM is mainly composed of type I collagen, and has good biomedical scaffold structure, mechanical strength, biodegradability and biocompatibility, which is conducive to cell infiltration, adhesion and growth. Its good biological activity has been verified in the rat skin wound repair model, which can induce the expression of biological factors related to skin repair, and also play a very good role in promoting skin wound healing. It is a very good biomedical tissue engineering scaffold material and has a good application prospect.

## Data Availability

The original contributions presented in the study are included in the article/Supplementary Material, further inquiries can be directed to the corresponding author.
